# The carbon emissions of writing and illustrating are lower for AI than for humans

**DOI:** 10.1038/s41598-024-54271-x

**Published:** 2024-02-14

**Authors:** Bill Tomlinson, Rebecca W. Black, Donald J. Patterson, Andrew W. Torrance

**Affiliations:** 1 Department of Informatics, University of California, Irvine, Irvine, CA 92697 USA; 2 School of Information Management, Victoria University of Wellington-Te Herenga Waka, Wellington, 6140 New Zealand; 3 Department of Mathematics and Computer Science, Westmont College, Santa Barbara, CA 93108 USA; 4 School of Law, University of Kansas, Lawrence, KS 66045 USA; 5 Sloan School of Management, Massachusetts Institute of Technology, Cambridge, MA 02142 USA

**Keywords:** Computer science, Climate-change mitigation

## Abstract

As AI systems proliferate, their greenhouse gas emissions are an increasingly important concern for human societies. In this article, we present a comparative analysis of the carbon emissions associated with AI systems (ChatGPT, BLOOM, DALL-E2, Midjourney) and human individuals performing equivalent writing and illustrating tasks. Our findings reveal that AI systems emit between 130 and 1500 times less CO2e per page of text generated compared to human writers, while AI illustration systems emit between 310 and 2900 times less CO2e per image than their human counterparts. Emissions analyses do not account for social impacts such as professional displacement, legality, and rebound effects. In addition, AI is not a substitute for all human tasks. Nevertheless, at present, the use of AI holds the potential to carry out several major activities at much lower emission levels than can humans.

## Introduction

Artificial intelligence (AI) has made rapid advancements in the past several years, with applications in a wide range of domains such as healthcare^1^, finance^2^, transportation^3^, and environmental conservation and sustainability^4^. These advancements have enabled AI to augment or even replace human capabilities in various areas, including problem-solving, decision-making, and some creative tasks. However, alongside the growing adoption and integration of AI into diverse sectors, concerns have emerged about the technology’s detrimental impact on the environment, particularly with regard to the energy consumption and resources required to develop, train, and maintain AI models, as well as accompanying greenhouse gas emissions (e.g.,^5,6^). For instance, the training of GPT-3, one of the most powerful and widely deployed AI systems to date, generates carbon emissions equivalent to the lifetime impact of five cars^7^.

Several of the skills that AI is being trained to execute—such as the ability to write or to create images—are activities that previously were almost exclusively the domain of humans. In this article, we analyze the environmental impact of several AI systems in relative terms, comparing their emissions to those of humans completing the same task. Specifically, we focus on the tasks of writing and illustration. By comparing the environmental impact of these tasks when completed by AI versus humans, we highlight the substitutability between humans and AI, and demonstrate that, while AI has substantial environmental costs, at present these costs are typically far lower than for a human completing the same task.

Previous research efforts have sought to compare the efficacy and/or impact of technological systems to those of humans. For example, Hagens^8^ offered multiple comparisons, such as that the work potential in one barrel of oil is equivalent to 11 hours of human manual labor, or that an electric cow-milking machine cuts the human effort required by a factor of 10 (at the cost of additional electricity being needed). Similarly, Brand^9^ compared the carbon footprint of walking and driving in cars. However, this article is the first time we are aware of where researchers have compared the carbon footprint of AI to that of humans.

This research seeks to address the UN Sustainable Development Goals, in particular Goal 12 (“Ensure sustainable consumption and production patterns”) and Goal 13 (“Take urgent action to combat climate change and its impacts”)^10^. It addresses Goal 12 via a reduction in Indicator 12.2.1 (“Material footprint, material footprint per capita, and material footprint per GDP”), since the energy used by either human or AI writers impacts their natural resource footprint. It addresses Goal 13 via a reduction in indicator 13.2.2 (“Total greenhouse gas emissions”), since the use of energy for human and AI writing lead to different levels of greenhouse gas emissions. Given the prevalence of writing and illustration in so many different aspects of corporate activity, governmental affairs, educational processes, and many other domains, we see even small changes in the environmental impact of these activities as being important. And here we present evidence not of a small change, but of one with the potential for a hundred- or even thousand-fold reduction in impact for portions of common human activities such as writing.

We recognize that these findings are not generalizable to all contexts. While AI use may be beneficial in some writing and illustration contexts, not all activities lend themselves to AI intervention. In fact, AI and humans cooperating on tasks may remain the best approach in many fields. In addition, these findings are based on the current state of AI and human activity; future changes in technology and society will likely change the environmental impact of both AI^11,12^ and that of humans^13^. There are also other complicating factors that need to be considered, such as professional displacement, legal use of training materials, and rebound effects. Nevertheless, the findings presented here suggest that concerns about the emissions generated by AI systems should be tempered by recognition that, even relying on cautious assumptions, humans produce far more emissions when engaging in some of the same tasks. While AI is often portrayed as an environmental threat to humanity, in this respect, at least, it may offer us valuable assistance.

In summary, this article contributes a comparison of the carbon emissions of humans and AI systems for the tasks of writing and illustrating, finding that AI has far lower emissions than humans at the same task. This contribution lays the groundwork for broader usage of AI in creative tasks. While the carbon emissions of humans and AI will certainly change over time, and such emissions are just one form of environmental impact (albeit likely the most important one with regard to climate change), we nevertheless find that this result has broadened our own willingness to utilize AI support in writing. We encourage others to use AI to support their own endeavors as well. At least based on the carbon emissions, using AI writing and illustration support is likely to be less environmentally impactful than writing equivalent text oneself.

## Methods

In this study, we conducted a series of numerical analyses to assess the environmental impacts of modern AI systems and humans, focusing on the tasks of writing and illustration. Our methodology consisted of a range of different elements. In line with best practices in life cycle assessment^14^, we engaged with the following four stages: goal and scope, inventory analysis, impact assessment, and interpretation.

### Goal and scope

The goal of this research effort is to compare AI writing and illustration with human writing and illustration. As will most life cycle assessment processes, it is impossible to include every item and process that has some bearing on the phenomena being studied (e.g., the food eaten by the instructor who taught one of the software engineers about AI, or the car driven by the English teacher who taught the person to write). Therefore, it is necessary to define the scope of the study.

For this study, we included the hardware and energy used to provide the AI service, but not the software development cycle or the software engineers and other personnel who worked on the AI. This choice is analogous to how, with the human writer, we included the footprint of that human’s life, but not their parents.

To ensure a comprehensive analysis, we selected four AI systems with different capabilities and energy requirements, including ChatGPT, BLOOM, DALL-E2, and Midjourney. These systems were chosen to represent a range of AI technologies, from natural language processing to image generation.

We considered the energy consumption and carbon emissions associated with human activities involved in writing and illustrating tasks. In particular, this assessment included factors such as the annual energy footprint of residents of various regions.

### Inventory analysis

Here we summarize the material and energy flows of the AI systems. While each AI system has different specific processes, in the broadest terms, a large dataset of training data is processed by a group of computer chips (here, GPUs). From this training process, an AI system is created that can answer many different queries as a result of a single act of training. Hence, the core structure of the analysis we conducted was to measure the total impact of the training process and divide it by the number of queries per training process, and then add it to the impact of the individual query

We also calculated the embodied energy in the devices used for both training and operation, as well as the decommissioning/recycling of those devices; however, as we discuss later, these additional factors are substantially less salient than the training and operation.

For the human writing process, we looked at humans’ total annual carbon footprints, and then took a subset of that annual footprint based on how much time they spent writing.

### Impact assessment

The results described below represent the impact assessment. We gathered previously published data from a range of sources, including peer-reviewed articles, government reports, and databases, to obtain information on the energy consumption, and carbon emissions associated with AI systems and human activities.

### Use of AI

As part of this research, we utilized ChatGPT (Jan 9 and Jan 30 versions of GPT-3 and March 14 version of GPT-4) to support the drafting and editing of sections within this article. Nevertheless, the core scientific work, including data analysis, calculations, and conclusions, was carried out by the authors. The authors carefully edited all AI-generated text to ensure that the quality of writing remained high. As recommended by Nature’s guidance, ChatGPT was not included as an author on this work^15^.

To guarantee the integrity and originality of our work, we ran the text through TurnItIn plagiarism detection software. This step ensured that ChatGPT did not inadvertently introduce plagiarism or violate copyright, maintaining the ethical and legal standards of academic research. This process is aligned with established best practices for scholarly writing with AI^16^.

Our usage of AI also aligns with the findings of this study, since incorporating AI into the writing process can be an environmentally sound decision when managed responsibly.

## Results

In this section, we present the results of our numerical analyses, which offer a comparison of the environmental impacts associated with AI systems and human activities in the context of writing and illustration tasks.

### Writing: AI vs. human

#### AI writing

While it can be difficult to define the scope of the problem when calculating the emissions produced by an AI system^5^, two major components of that impact are the training of the model (a one-time cost that is amortized across many individual queries) and the per-query emissions. To offer two data points on the environmental impact of training models, training GPT-3 (the system on which the popular ChatGPT chatbot is based^17^) produces approximately 552 metric tons CO2e^11^. Training BLOOM, a model slightly larger and substantially more energy-efficient than GPT-3, produces 50.5 metric tons of CO2e^12^.

Apart from the amortized emissions associated with training, each AI system query also contributes to the overall emission footprint. An informal online estimate for ChatGPT indicates that it produces 0.382 g CO2e per query^18^, based on 3.82 metric tons CO2e per day divided by 10,000,000 queries per day. In comparison, a deployment of BLOOM generated 1.5 g per query (340 kg CO2e divided by 230,768 queries)^12^.

Assuming that ChatGPT undergoes a full re-training of the model once per month and continues with an estimated 10,000,000 queries per day, the 552 metric tons divided by 300,000,000 queries equates to 1.84 g CO2e per query for the amortized training cost. Consequently, the combined impact of training and operation for ChatGPT amounts to approximately 2.2 g CO2e per query. For BLOOM, assuming a similar level of usage and frequency of retraining as for ChatGPT, the per-query impact of training is 0.10 g CO2e, while the per-query operational cost is 1.47 g. This results in a total emission of 1.6 g per query for BLOOM.

We also calculated the embodied energy of the chips used to train the model and of the server used to deliver the query, and the end-of-life recycling of those devices, but all of these factors were 2–5 orders of magnitude smaller than the training and per-query emissions. The embodied energy footprint of an A100 GPU is 150 kg CO2e^12^. The energy footprint for recycling an equivalent device is 1 kg CO2e^19^. Training BLOOM requires 433 MWh of energy, and training GPT-3 requires1287 MWh^12^. Approximating that a GPU lasts 1.5 years before becoming obsolete^20^, the amortized training cost per query for BLOOM is 0.00004 g CO2e, and for GPT3 is 0.0001 g CO2e. Similarly, the embodied energy of the server used to conduct the query is approximately 2500 kg CO2e^12^. Since, based on our own measurement, a GPT3 query takes approximately 3.8 s per page of text (4.4 s for ChatGPT to produce 292 words), the embodied energy of the server per query is 0.03 g CO2e. These values are several orders of magnitude lower than the energy footprint of training and conducting the query.

These figures illustrate that the impact of an AI query, encompassing both amortized training and the query itself, is on the order of a few grams CO2e.

#### Human writing

To calculate the carbon emissions associated with human writing, we first examine the writing speed and productivity of human writers. An article in The Writer magazine states that Mark Twain’s output, which was roughly 300 words per hour, is representative of the average writing speed among authors^21^. Therefore, we use this writing speed as a baseline for human writing productivity.

To calculate the carbon footprint of a person writing, we consider the per capita emissions of individuals in different countries. For instance, the emission footprint of a US resident is approximately 15 metric tons CO2e per year^22^, which translates to roughly 1.7 kg CO2e per hour. Assuming that a person’s emissions while writing are consistent with their overall annual impact, we estimate that the carbon footprint for a US resident producing a page of text (250 words) is approximately 1400 g CO2e. In contrast, a resident of India has an annual impact of 1.9 metric tons^22^, equating to around 180 g CO2e per page. In this analysis, we use the US and India as examples of countries with the highest and lowest per capita impact among large countries (over 300 M population).

In addition to the carbon footprint of the individual writing, the energy consumption and emissions of the computing devices used during the writing process are also considered. For the time it takes a human to write a page, approximately 0.8 h, the emissions produced by running a computer are significantly higher than those generated by AI systems while writing a page. Assuming an average power consumption of 75 W for a typical laptop computer^23^, the device produces 27 g of CO2e^24^ during the writing period. It is important to note that using green energy providers may reduce the amount of CO2e emissions resulting from computer usage, and that the EPA’s Greenhouse Gas Equivalencies Calculator we used for this conversion simplifies a complex topic. However, for the purpose of comparison to humans, we assume that the EPA calculator captures the relationship adequately. In comparison, a desktop computer consumes 200 W, generating 72 g CO2e in the same amount of time.

**Figure 1:**
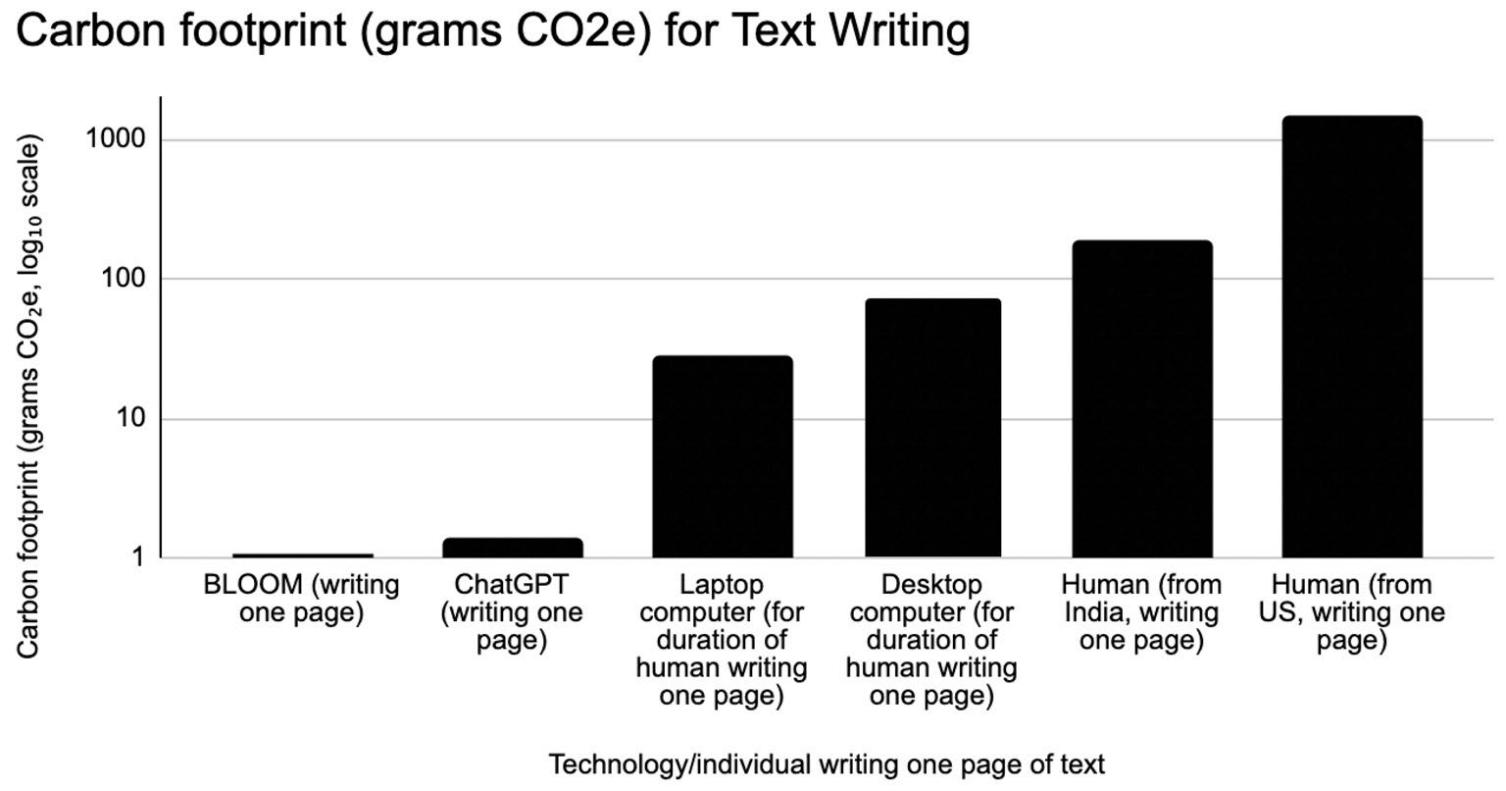
This figure compares the CO2e emissions of AI and humans engaged in the task of writing one page of text. AI writing (via BLOOM or ChatGPT) produces 130–1500 times less CO2e per page than a human author. AI also produces substantially less CO2e than the computer usage to support humans doing that writing.

#### Comparison

Figure 1 compares several variations of authorship: BLOOM is 1400 times less impactful, per page of text produced, than a US resident writing, and 180 times less impactful than a resident of India writing. ChatGPT is 1100 times less impactful than a US resident writing, and 130 times less impactful than a resident of India writing. Assuming the quality of writing produced by AI is sufficient for whatever task may be at hand, AI produces less CO2e per page than a human author (We note that just the time spent by the human writing the query and waiting for the query to be handled by the server has a far greater footprint than the AI system itself. If a person takes 1 min to write a query, and needs to wait 4 s (0.07 min) for the query to be handled: at 15 metric tons CO2e per year for a US resident, 1.06 min has a footprint of 30 g CO2e, approximately 15 times greater than the AI itself. In addition, we note that there is significant complexity to writing processes: both human- and AI-produced text will likely need to be revised and rewritten based on the human authors’ sense for how effectively the text expresses the desired content. Since this revision process exists in both human and AI-assisted writing, we feel it is beyond the scope of this analysis. Future work could assess whether the editing process tends to be more iterative and time-intensive in human writing with or without AI.).

Authorship does not exist in a vacuum, and any accounting for the return on energy expenditure is confounded by the impact to the rest of the network in which it is embedded. For example, successful AI deployments may beget more costly models in the future, more frequent prompts by users, or more costly training schedules. On the other hand, human authorship may implicitly be training for other kinds of productive human work that would be lost in the face of the proliferation of AI writing. The freed human time may also incur new unexpected environmental costs.

### Illustration: AI vs. human

In this section, we examine the environmental impact of AI and human illustrators in the context of creating visual content.

#### AI illustration

In this section, we analyze the environmental impact of AI-generated illustrations. We examine two prominent AI image generation engines: DALL-E2 and Midjourney. DALL-E2 is based on an underlying GPT-3 engine (similar to ChatGPT above), and Midjourney is based on a system called stable diffusion.

Given their shared reliance on GPT-3, we estimate that DALL-E2’s footprint is similar to the footprint of ChatGPT calculated above: 2.2 g CO2e per query. To estimate the impact of Midjourney, we take a different approach, based on statements made by Midjourney’s CEO David Holz. Holz stated, with regard to Midjourney’s computer usage, that “[e]very image is taking petaops ... So 1000s of trillions of operations. I don’t know exactly whether it’s five or 10 or 50. But it’s 1000s of trillions of operations to make an image... [W]ithout a doubt, there has never been a service before where a regular person is using this much compute”^25^.

AI data centers, such as Google’s Compute Engine^26^, often run on Nvidia A100 GPUs^27^. These GPUs can process 1.25 petaoperations per second while using 400 W of electricity^27^. In the largest-emissions scenario (from Holz’s comments), generating an image requires 50 petaoperations; therefore, the AI would need to run on that device for 40 s. This work would require 4.5 Wh to process, emtting approximately 1.9 g CO2e^24^.

#### Human illustrator

Estimating the environmental impact of a human illustrator is challenging for several reasons: there is a wide range of time that it may take for a human illustrator to produce an illustration, depending on the complexity of the work, the artist’s expertise, and the specific requirements of the project.

To arrive at an estimate for how long it takes, on average, we combined the average cost for an illustration project ($200^28^) and the average hourly rate of pay for an illustrator ($62.50/h^28^) Based on these figures, we propose that 3.2 hours per illustration is a viable estimate for a professional illustrator producing a commercial piece of work based on a provided specification, across a wide range of styles and formats.

Since the environmental footprint for a US resident is approximately 15 metric tons CO2e per year^22^, we calculate that the carbon footprint for a US-based illustrator is approximately 5500 g CO2e per image. For a resident of India, by comparison, the impact would be approximately 690 g CO2e per image^22^.

As an additional point of comparison, we also calculate the carbon footprint of the devices that human illustrators may use while working, using similar calculations as in the writing comparison above. The carbon footprint for a laptop operating for the duration of a human illustrator creating an image (3.2 h) is 100 g CO2e. The footprint of that duration for a desktop computer is 280 g CO2e.

These estimates may vary depending on factors such as location, individual energy consumption, and illustration complexity. Additionally, as with AI-generated text, the environmental impact of human illustrators should be considered in the context of the broader creative process and the potential for collaboration between humans and AI systems to optimize both artistic quality and environmental sustainability. Nevertheless, this calculation provides a rough estimate of the impact of human illustration.

**Figure 2:**
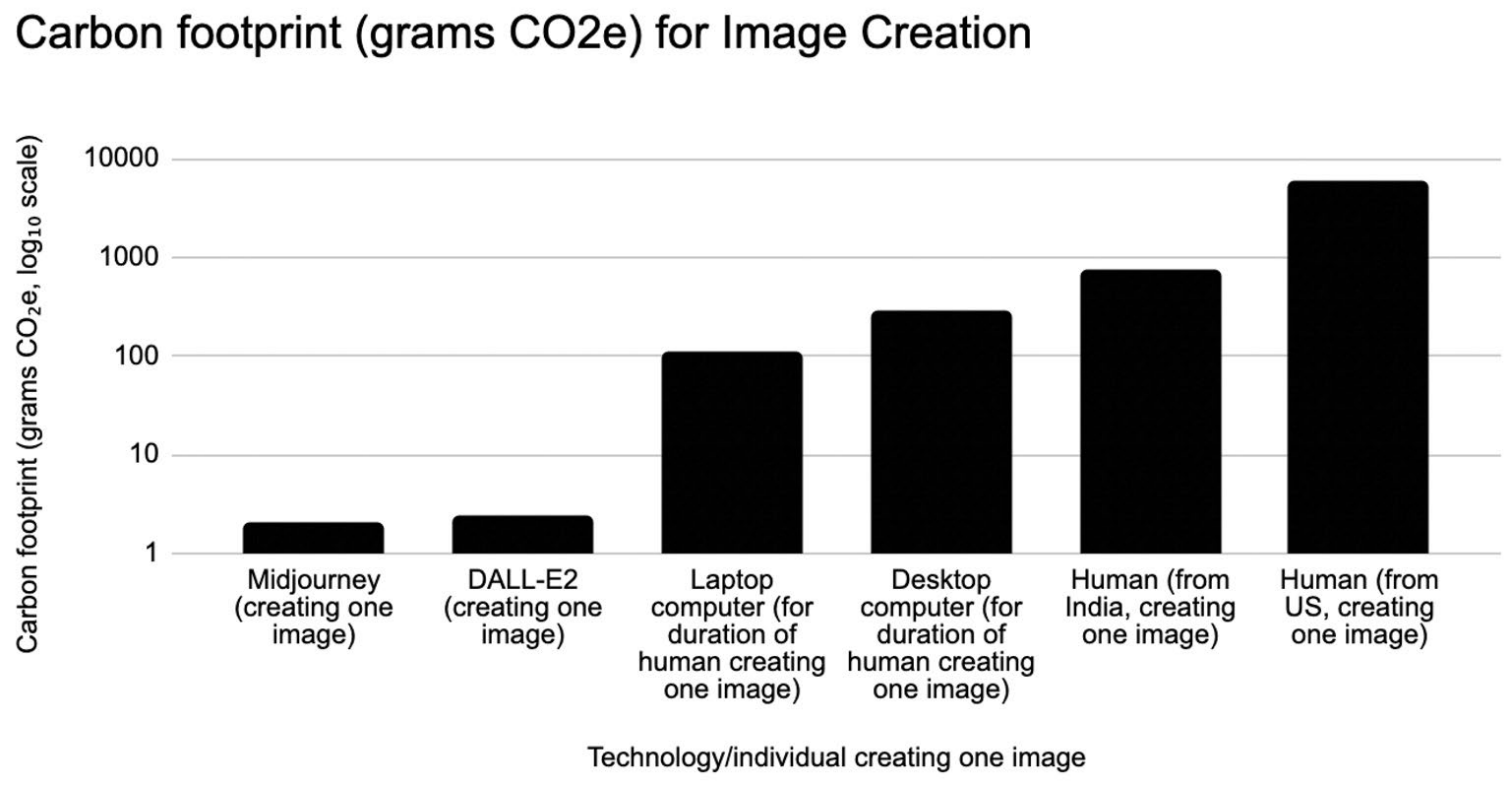
This figure compares the CO2e emissions of AI and humans engaged in the task of creating one image. AI image creation (via DALL-E2 or Midjourney) produces 310–2900 times less CO2e per image than human creators. AI produces many times less CO2e than computer usage to support humans making images.

#### Comparison

Figure 2 shows that DALL-E2 emits approximately 2500 times less CO2e than a US-based artist, and approximately 310 times less than an India-based artist. Midjourney emits approximately 2900 times less CO2e than a US artist, and 370 times less than one based in India. Here, as with the writing analysis above, both laptop and desktop usage while supporting a human drawing an image would themselves be many times more impactful than the AI systems as well.

## Discussion

The findings above demonstrate that the environmental footprint of AI completing two major tasks is substantially lower than that of humans completing those same tasks.

### Benefits and drawbacks

While the environmental footprint of AI may be lower than that of humans for certain tasks, there are other important factors that may influence AI’s overall impact on the world. Many relevant issues have been discussed elsewhere^29,30^; here, we touch on a few items of most relevance to AI for writing and illustrating.

In the domains addressed by this article (writing and illustrating), it is likely that AI will displace human workers in relevant industries as AI technology becomes more advanced. And, if the past is any indicator, professional displacement may lead to job losses and reduced income. The displacement of jobs by technology has been amply studied, e.g.,^31^, as has displacement by AI in particular^32^. Job displacement is deeply problematic not only to those displaced, but to society at large, as it can disrupt the economic and social stability of entire geographic regions.

On the other hand, the development of AI has the potential to create jobs as well. These jobs could be meaningful and well-compensated replacements for those AI displaces, or they could be demeaning and/or involve low pay. For example, OpenAI, the creators of ChatGPT, outsourced work to a Kenyan company where workers were employed to label specific instances of toxic online content, including content many would likely find disturbing or distasteful, described as “text [that] appeared to have been pulled from the darkest recesses of the internet”^33^. Analogous displacements of workers took place during the Industrial Revolution and with the various technological revolutions accompanying the rise of digital technologies. While these displacements necessarily cause changes in the job industry, historically such technological shifts have given rise to new forms of employment to replace those lost.

There are also legal issues that are pertinent to the use of preexisting text, images, or sounds as training sets for AI. The legality of using preexisting material is particularly salient when training sets include copyrighted material, because use of such material may infringe, potentially posing a risk of legal exposure for individuals who work with such AI systems. Perhaps “fair learning” will one day be recognized as a type of fair use that involves the transformation of copyrighted materials for educational purposes. However, at present, it remains unpredictable how courts will decide such a dispute. There is a class action lawsuit against the AI company Midjourney currently pending on this topic^34^ that may provide precedent in this legal domain. Were Midjourney to be held liable for impropriety in using copyright works owned by others, the generous statutory damages scheme available to the plaintiffs could be ruinous for that particular company, while, more generally, chilling or inhibiting innovation in AI. On the other hand, if AI use of copyrighted material as training sets is held to be permissible, this will likely have the effect, within the current patent system, of spurring advances in AI. Another outcome could be the rise of companies acquiring vast sets of training data. While these legal issues are not necessarily intractable, they nevertheless represent an important point of contention over the future of such AI systems.

Additionally, as AI technology becomes more efficient, it is possible that such efficiency will lead to an increase in the demand for AI-produced goods and services, which could lead to further increases in resource use and pollution via rebound effects^35^. The broadening of use cases for AI, and the proliferation of ways that AI could impact each use case (e.g., ubiquitous personalization of content) could lead to potentially far greater demand for energy than occurs at present. As such, while the impact of AI is currently far less than humans in the tasks described above, it is important to maintain vigilance in this domain to avoid runaway resource use. At the same time, it is possible that advances in the efficiency and specificity of AI could further decrease its environmental impact compared to human impacts from equivalent activities. Such an increasing environmental advantage could argue in favor of accelerating applications of AI. In either scenario, vigilance and adaptation are vital. And, whether the footprint of AI goes up or down, we support the call for disclosure of energy consumption to whatever degree possible across AI use cases^36^.

Despite these current and potential future forms of societal transformation and harm, profound benefits to society could accrue through the use of AI. Such systems could enable the development of new approaches to sustainable futures^36^; they could lead to benefits in medicine^37^; and they could improve human educational systems^38^. We argue that these and other benefits of AI offset the potential harms such systems may entail. And most relevant to the findings of this paper, AI can potentially do so with substantially lower carbon emissions.

### Human/AI collaboration

We argue that the most beneficial and efficient use of both AI and human labor is via collaboration between the two types of entity, taking advantage of their respective strengths. For example, in this article, we began with a draft written by an AI to bootstrap the effort, but the authors have edited it so thoroughly that the AI text is unrecognizable. (We acknowledge this use of AI for two reasons; first, it is required by the submission guidelines and second, and perhaps more importantly, starting with AI was a more energy efficient way to achieve a high quality final product.) Similarly, a human illustrator may choose to work with an AI in the early stages of an interaction with a client, to give them a sense of the broad range of possibilities available to them, and then complete a human-created illustration for the client only at the last stage. Such a hybrid approach could allow for more rapid and more efficient coalescing of understanding between the client and the human illustrator, while also producing a final product that has the excellence and polish of a human-produced piece of work. (For example, unlike many AI-produced images, the human hands won’t be uncannily misrepresented^39^). Hopefully such collaborative processes may address a range of concerns about AI-generated content^40^.

In sum, due to its substantially lower impact than humans at at least two important tasks, AI can play an important role in various sectors of society without, at present, running afoul of problematic carbon emissions. While the carbon footprint of AI is nontrivial, the footprint of humans doing the same work is far greater, and should not be discounted in the assessment of AI.

## Limitations

The results for each specific task reflect an array of assumptions about the nature of these tasks and the people and AIs engaged in such tasks. For example, writing an in-depth, heavily-referenced, original article on a niche scientific topic is currently beyond the capabilities of an AI, and therefore is a context where human effort is more efficient than AI effort (since the AI cost for such a task is effectively infinite, at present). In the domain of illustration, drawing a stick figure is likely faster for a human than an AI at present (and therefore may have lower emissions due to dramatically lower speed), whereas the reverse is true for a complex illustration such as one resembling an oil painting. Nevertheless, despite these specific regions of the task-space where humans have lower emissions, the data presented in this paper suggest that, overall, the use of AI can significantly reduce the carbon footprint of certain tasks when compared to equivalent human activity.

These findings are also based on the current state of AI and human activity; future changes in technology and society will undoubtedly change the relative environmental impact of AI as well. For example, as evidenced by the order-of-magnitude difference in emissions from training GPT-3^11^ vs. training BLOOM^12^ despite similar sized training data, algorithmic advances may profoundly reduce the footprint of AI systems, as has already been hypothesized^11^. Alternatively, advances may improve the performance of AI, but at the cost of dramatic increases in energy use and accompanying emissions. For example, the possibility of ubiquitous personalization of AI content, in which all media consumed by everyone on earth—every book, every movie, every game, every educational worksheet—has been precisely tailored to that individual’s unique and evolving preferences, paves the way for vastly greater emissions footprints for future AI systems. Whether the net effect of increasingly efficient algorithms and larger training sets and deployment contexts will cause total energy use to increase or decrease over time remains to be seen.

Similarly, societal changes regarding the footprint of various human societies may also change the ratio between AI and human activity. For example, the per capita impact of a human in the US has been mostly falling since it peaked in the 1970s^22^, and the per capita impact of a human in India has been rising almost continuously since the 1940s^22^ (although the impact of a resident of India is still only one seventh the impact of a US resident). These trends may continue, or may be altered by social and/or technical changes. Either way, they are highly likely to affect the human side of the AI/human ratio of the environmental costs related to the activities addressed in this article.

## Conclusion

AI is poised to take over roles once thought to be solely the domain of humans—those requiring creativity and the ability to integrate across multiple intellectual domains to synthesize concepts from each. In this study, we have compared the impact of AI to the impact of humans performing two tasks—writing and illustration—to highlight the role that AI is positioned to take in society, as AI transitions from digital tools of limited utility to more complex instruments with high generative capacity. We found that, for these two activities, at least, AI has a substantially lower carbon footprint than humans engaged in the same task. This study provides new insights on the relative environmental footprint of AI and humans, and it highlights the importance of considering the impact of AI relative to a human when evaluating its overall impact on the environment.

## Data Availability

All data and calculations can be found online at: https://doi.org/10.17605/OSF.IO/YHTMQ.
